# Modernizing the Institutional Review Board Process for Implementation Science – A Narrative Review of Barriers and Ethical Considerations

**DOI:** 10.1007/s11883-026-01454-8

**Published:** 2026-08-28

**Authors:** Abhishek Bazaz, Caroline Plott, Francoise A. Marvel, Allison W. Peng, Stacey Schott, Benjamin M. Scirica, Jessica M. Peña, Ankeet Bhatt, Laney K. Jones, Seth S. Martin

**Affiliations:** 1 Ciccarone Center for the Prevention of Cardiovascular Disease, Division of Cardiology, Johns Hopkins School of Medicine, Baltimore, MD USA; 2 TIMI Study Group, Division of Cardiovascular Medicine, Brigham and Women’s Hospital, Harvard Medical School, Boston, Massachusetts USA; 3 Dalio Institute of Cardiovascular Imaging, Department of Radiology, Division of Cardiology, Department of Medicine, Weill Cornell Medicine, New York, NY USA; 4 Department of Cardiology, Kaiser Permanente San Francisco Medical Center, San Francisco, California USA; 5 Amgen Inc, Thousand Oaks, CA USA

**Keywords:** Implementation science, Research to practice, Quality improvement, Institutional review board, Human research ethics, Research governance

## Abstract

**Purpose of Review:**

This review highlights the barriers and facilitators that implementation science studies face in the institutional review board (IRB) approval pathway and provides recommendations on how to potentially streamline this process.

**Recent findings:**

Significant gaps persist between evidence-based recommendations and real-world clinical practice. Implementation science projects investigate how to promote uptake of evidence-based and guideline-recommended practices. Regulatory ambiguity may arise because implementation science involves aspects of traditional research and quality improvement. Currently, there is a lack of well-delineated processes to determine the optimal review pathway for implementation science projects.

**Summary:**

Current research review pathways do not facilitate efficient approval for implementation science projects due to ambiguous IRB submission guidelines, limited exposure to implementation science, and recent integration of digital tools. In this review, we suggest further development of expedited review pathways for low-risk projects, creation of tools for researchers to determine IRB application category, and further investment in digital health-focused implementation science projects and approval pathways to prepare for the emerging wave of implementation science and maximize the positive impact on patient health outcomes.

## Introduction

Despite robust investments in healthcare-related research in the United States, significant barriers exist in translating evidence-based interventions into practice. Indeed, on average, it takes 15–17 years to implement scientifically backed findings into clinical practice [1, 2]. The reasons for this prolonged pipeline appear to be multifactorial and include (1) significant inertia to change clinical practice patterns, (2) rigid funding mechanisms that limit support for innovative approaches, and (3) prolonged and complex approval processes for implementation science research projects.

Implementation science is the study of methods that promote systematic uptake of established clinical guidelines and evidence-based interventions into routine healthcare practice [3, 4]. The efficiency of regulatory review of implementation science projects is a rate-limiting factor in the translation of evidence-based practices to patient care. Efficiency may be hampered by a lack of clarity in project classification and the review pathway. There is significant ambiguity between what qualifies as implementation science, traditional human subjects research, and quality improvement projects. Implementation science is often classified as human subjects research, but also may have features of quality improvement and tends to be more iterative and flexible, with a goal of improving practice that aligns with quality improvement. This narrative review, focused on the United States, examines institutional review board (IRB) considerations for implementation science projects, highlights the barriers and facilitators implementation science studies face as they are processed through the IRB approval pathway, and provides suggestions for streamlining this process.

## Defining and Comparing Human Subjects Research, Quality Improvement, and Implementation Science

According to the National Institute of Health, human subjects research involves either (i) the collection of information or biospecimen via interaction or intervention with an individual or (ii) the study, collection, use, or generation of private data or biospecimens [5, 6]. A key aspect that differentiates human subjects research projects is the higher risk level to participants. The studies involve investigational drugs or practices and thus confer greater risk to a study participant’s health. As a result, human subjects research projects require full IRB review and strict adherence to regulations to protect research participants. Additionally, human subjects research is differentiated by having a goal of establishing the safety and efficacy of novel treatments or interventions. Human subjects research projects are often clinical trials or other studies that randomize patients to different treatment groups, which can include an investigational drug vs. a placebo or drug(s) vs. other drug(s). Human subjects research projects may have aspects of quality improvement when they are a hybrid study design, this approach seeks to investigate both effectiveness and implementation. For example, a research study of patients with concomitant depression and heart failure sought to connect these participants with mental health treatment for their mood disorder. This study looked at both the outcomes from the implementation of an evidence-based algorithm to connect patients with depression care and investigated the real-world applicability concerns [7]. In this case, given that the project seeks to implement a new intervention that has not yet been proven efficacious, this project would likely need to be evaluated through the traditional human subjects research IRB pathway despite having components of an implementation science project [8].

In contrast to human subjects research projects, quality improvement studies focus on the optimization of clinical practices and assessment of productivity, safety, or patient experience. Compared to human subjects research projects, quality improvement studies are often done at a local level, completed in a shorter time period, and can be implemented more rapidly. Quality improvement studies use established standards of care, and thus participants are generally not exposed to significant risks to their health, assuming that there are no unintended interruptions in workflow. For example, in the study “Enhanced Safety and Efficiency of Ambulatory Cardiology Admissions: A Quality Improvement Initiative,” investigators analyzed whether a new closed-loop communication pathway and in-clinic patient monitoring system led to increased safety metrics and decreased time-to-admission from an ambulatory cardiology center [9]. No new test or treatment was involved, but rather a new workflow. As such, quality improvement studies are not subject to the same level of IRB oversight and regulations as human subjects research.

The Expert Recommendations for Implementing Change (ERIC) project addressed conceptual clarity and nomenclature of implementation science strategies, defined as “methods or techniques used to enhance the adoption, implementation, and sustainability of a clinical program or practice [5].” Implementation science serves as a bridge between evidence-based practice and the realization of that benefit at greater scale in routine clinical or public health practice. This is an essential step in the translation pipeline as it evaluates barriers and facilitators, demonstrates how an intervention or clinical practice can deliver benefit in real-world clinical practice, and enhances uptake of evidence-based care [10]. Ultimately, implementation science projects inform the choice of which evidence-based practices should receive the time and resources toward a full-scale deployment.

Implementation science incorporates components of both human subjects research and quality improvement studies, with the goal of increasing uptake of evidence-based practices [11]. Like human subjects research, implementation science studies often involve making changes to clinical care processes that directly affect patient care. However, unlike human subjects research, these changes are based on interventions that already have established safety and efficacy, with the goal of optimizing delivery of existing standards of care. Like quality improvement, the primary outcomes in implementation science focus on adoption, fidelity, feasibility, and sustainability of practice rather than on generating new evidence for clinical effectiveness. As a result, implementation science studies typically pose lower incremental clinical risk than human subjects research studies, as they do not introduce novel therapies or unproven exposures. Together, these features position implementation science as a pragmatic, lower-risk research paradigm designed to close the gap between evidence-based research and what is accomplished in routine clinical practice.

As a broad field, implementation science has features of both human subjects research and quality improvement initiatives, making the choice of IRB pathway challenging, but they each have distinct primary intents, scopes, regulatory pathways, and methodology (Table 1).

**Table 1:** Comparing human subjects research, quality improvement and implementation science

	Human subjects research	Quality improvement	Implementation science
Primary Intent	Contributing to the general body of evidence based practice knowledge	Improve local patient care	Promote uptake of proven clinical care
Methodology	Rigid protocol with set controls	Flexible and rapidly iterative protocol	Mixed flexibility of protocol
Intervention	Often unproven or experimental treatment	Usually process refinement with consideration of established standards	Strategy or strategies to increase uptake of guideline-recommended care
Risk	Can expose patients to risk associated with unproven medical treatment	Minimal risk to the patients outside of routine care/data safety	Usually minimal as treatments have already been established as ready for uptake by guidelines
IRB/Regulatory Oversight	Formal IRB approval	IRB exemption vs. quality improvement-specific accelerated IRB review	Mixed, depending on project may require formal IRB approval vs. quality improvement-specific pathway

## Lack of Implementation Science Specific IRB Submission Pathways

In the absence of a dedicated implementation science IRB review and the regulatory gray zone between traditional human subjects research and quality improvement, there is a misalignment for the most effective submission pathway for implementation science. Researchers may attempt to apply to the IRB through an expedited quality improvement pathway. However, depending on the IRB categorization of the project, it may be rerouted through the regular human subjects research IRB, leading to significant project delays.

## Approval Barriers to Implementation Science Projects

Given its investigatory nature and frequent involvement of human subjects, implementation science projects often undergo IRB review, which can demand significant time and capital investment. However, implementation science protocols involve deploying tools and practices that have previously been studied through the traditional human subjects research route and focus more on testing strategies for uptake; therefore, the risk to participants is often very low [6, 11]. It is both logical and advantageous for IRBs to adopt more streamlined review pathways for low-risk implementation science studies. Reducing unnecessary administrative burden while maintaining appropriate ethical oversight would facilitate more rapid translation of proven clinical evidence into practice, ultimately advancing the goals of improved patient care. To our knowledge, there are no widely adopted formal IRB review pathways specialized for implementation science projects. Therefore, during the IRB application process, implementation science researchers must determine whether their project falls under human subjects research or quality improvement to inform whether they must proceed through the conventional IRB process. The traditional human subjects research process is time-intensive, and if a project is initially classified as quality improvement but later considered human subjects research by an IRB committee, this often requires revision and resubmission of the application, creating further delays. Implementation science has historically received less funding than discovery research, with funding discrepancies of up to 100-fold [12, 13]. A shorter, less expensive IRB process would enable a more sustainable implementation science ecosystem.

## Decision-Making Tools to Differentiate Human Subjects Research & Quality Improvement

**Fig. 1:**
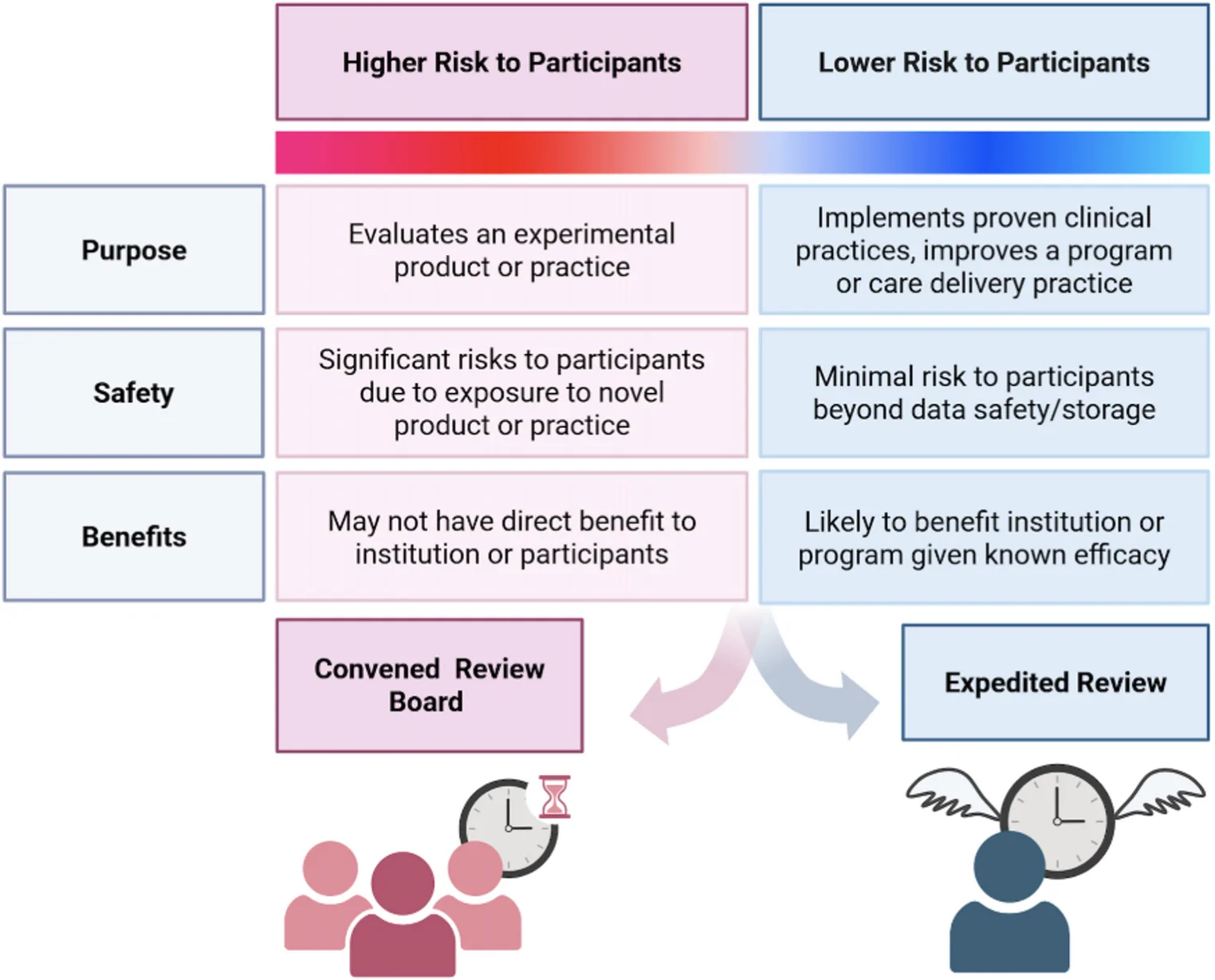
Determining the proper IRB pathway for implementation science projects based on risk, purpose, safety, and benefits [6].

One approach to distinguishing between human subjects research and quality improvement projects is providing researchers with a decision-making tool to classify the project and guide the appropriate submission pathway. For example, one institution’s IRB uses a checklist that allows users to tally features of their study as human subjects research versus quality improvement [6]. Below, we outline common areas of experimental design that these checklists highlight to differentiate quality improvement and human subjects research, as well as how implementation science studies often occupy a grey area in each section. Despite these resources, the categories remain subjective and not completely dichotomous. Given that these tools are not standardized, and decision-making pathways are nuanced, the threshold for submitting a project for human subjects research designation to the IRB submission could vary across institutions. Even if studies meet criteria outlined in IRB checklists as quality improvement, this does not guarantee the study will ultimately be determined to meet quality improvement criteria when formally evaluated by the IRB committee. To address this issue, we propose the development of standardized and transparent decision-making tools utilizing some of the categories outlined below for implementation science researchers to determine the proper pathway to apply (Fig. 1).

### Purpose of the Research or Initiative

For many implementation science projects, it is difficult to classify whether the primary purpose is local improvement, generalizable discovery, or both. For example, in the multisite trial “Improving Evidence-Based Care of Heart Failure in Outpatient Cardiology Practices,” reportedly sites were allowed to participate either if they obtained full IRB approval or a waiver; however the study does not provide specifics regarding which sites were able to obtain waivers and why [14].

### Starting Point

Human subjects research generally tests a hypothesis that is independent of routine clinical care. This involves testing a treatment that would otherwise not be given to patients to generate new knowledge. Quality improvement involves an iterative and evaluative process to impact clinical care and delivery. The findings of these studies are used to inform and iterate on current care. Implementation science may be considered a hybrid of these approaches. For example, current guidelines recommend less than a 90-minute delay between symptom onset of myocardial infarction and percutaneous coronary intervention [15]. The study “Strategies for Reducing the Door to Balloon Time in Acute Myocardial Infarction” investigated methods to help hospitals meet this guideline [16]. Some of these interventions included having emergency medicine physicians directly activate the catheterization laboratory or having a cardiologist always on site. While these interventions were outside routine operations, they did not directly involve the clinical care being administered to the patients.

### Design

Quality improvement and human subjects research projects can overlap in study design. Human subjects research is more likely to follow a predefined protocol with fixed endpoints, often involving multicenter collaboration and external funding. Generally, quality improvement tends to rely on an adaptive, iterative approach, typically within a single site and without external funding (though this is not a defining characteristic). Implementation science studies’ designs may evolve, like quality improvement, but are also prospectively planned and rigorously evaluated like human subjects research. The presence of adaptive elements within a formally funded, protocol-driven study blurs categorical boundaries. Furthermore, implementation science projects should still be held to high study design standards, including considering equity. These indirect risks are considerations to avoid perpetuating disparities [10].

### Benefits

The primary goal of human subjects research is to guide future care practices by establishing statistically meaningful differences between treatment and placebo. While ultimately this knowledge hopefully will benefit patients, direct benefit to participants is a welcome possibility but not the principle goal of the work. Indeed, participants in randomized control trials that are human subjects research may have a “therapeutic misconception,” which is the mistaken belief that the primary purpose of the research study is to provide individualized clinical care or direct therapeutic benefit, despite the random possibility that they could be in the placebo group and not receiving any therapy. Quality improvement projects, on the other hand, are intended to produce immediate improvements in care delivery or outcomes to all participants as well as to produce knowledge about care practices generally.

### Risks

Human subjects research can introduce risks beyond standard clinical care, whereas quality improvement generally poses minimal risk, limited mainly to issues of privacy or data confidentiality. Implementation science projects typically introduce lower clinical risk because they deploy practices that are already evidence-based and considered standard of care. However, implementation science may still introduce system-level risks such as workflow disruptions, unintended care delays, and standard risks of introducing new care practices. It is often challenging to quantify the severity of these indirect risks, making risk-based classification more subjective than in traditional human subjects research. These risks may be mitigated by tracking workflow and timing of care, and making iterative changes.

### Endpoints

Generally, human subjects research seeks to answer a specific research question, using predefined primary and secondary endpoints, often related to clinical efficacy or safety. Quality improvement focuses on measuring and improving performance relative to established standards or benchmarks. Implementation science focuses on how well evidence-based interventions are adopted, delivered, and sustained in real-world settings, rather than clinical efficacy alone. These endpoints are often assessed using established implementation frameworks. A commonly used framework to evaluate the impact of interventions is the RE-AIM (reach, effectiveness, adoption, implementation, and maintenance) framework [17]. The categories provide metrics to gauge intervention success on an individual and system level while also evaluating sustainability. While these endpoints resemble quality improvement metrics, when they are analyzed for general scientific inference, they contribute to ambiguity in the IRB project classification.

### Analysis

Analysis strategy is another area with significant overlap between human subjects research and quality improvement projects. In broad strokes, human subjects research uses formal statistical testing to prove or disprove hypotheses and quality improvement uses data to assess progress toward improvement goals or best practices, however this analysis may also include formal statistical testing. Thus, the analysis strategy is not a reliable way of differentiating between these types of research.

### Dissemination

Both human subjects research and quality improvement projects may be disseminated either locally or to a broader audience through publication. Human subjects research is explicitly designed for dissemination beyond the local setting, typically through peer-reviewed publication and broader scientific communication. Quality improvement dissemination can be primarily local, aimed at sustaining improvements within an institution, though successful initiatives may be shared externally. Implementation science explicitly focuses on dissemination and scale-up, as its central goal is to inform how evidence-based practices can be adopted across diverse settings. As a result, implementation science aligns closely with human subjects research in dissemination intent even when its operational features resemble quality improvement.

## Navigating Approval for Digital Health Focused Implementation Science Projects

Digital health is the intersection of healthcare and technology, aiming to use digital tools, data, and communication technologies to deliver, manage, and improve health. This area of work is increasingly a focus of implementation science, both to improve clinical care and facilitate study design. Indeed, even Food and Drug Administration Commissioner Marty Makary in January 2026 announced a shift towards deregulation of wearable health technology to support earlier access to innovative and lifestyle-focused artificial intelligence-enabled wellness/health wearable technologies [18]. There is substantial interest in understanding the validity and clinical utility of digital health products built for the healthcare space that use apps, wearables, and/or artificial intelligence algorithms [19]. There are many examples of implementation science research that focus on digital health interventions. For example, the CONNECT-HF trial investigated whether the use of digital health applications was associated with improved clinical outcomes among patients with heart failure with reduced ejection fraction. The digital health applications provided heart failure education content, tracked biometric data, and sent regular medication reminders. Matching digital users with nonusers from the usual-care group, the study found that the use of digital applications was associated with higher HF quality-of-care scores, including use of GDMT, and more favorable composite clinical outcomes inclusive of rehospitalization or all-cause mortality [20].

While there are potential benefits of digital health projects, as with all areas of research, these projects carry unique risks. IRBs must evaluate not only the usual human-research risks, but also novel risks associated with digital health: data security and integrity, third-party data usage, terms of service, device malfunction or unintended clinical consequences, algorithmic bias, or unintended decision-support effects. Therefore, use of digital health in implementation science may increase the complexity of IRB review and tend to push a project towards human subjects research. Beyond the primary IRB committee, use of digital health may require review by data-trust or privacy offices/committees, clinical engineering or biomedical informatics governance, cybersecurity, compliance offices, vendor contract committees, legal offices, and sometimes institutional digital health committees. The integration of artificial intelligence, machine learning components and digital data pipelines further complicates oversight. The Subcommittee on Institutional Review Boards (SACHRP)’s recent guidance for IRBs highlights the challenges posed by artificial intelligence and machine learning use in human subjects research, including algorithmic bias, large-scale data aggregation, and the need for transparency around model training and deployment [21].

Implementation science projects involving digital health often engage large volumes of personal health and behavioral data, involve third parties, and may influence clinical workflows in ways that go beyond traditional implementation science interventions. The World Health Organization has provided guidelines for health stakeholders involved in digital health to guide development and resource investment in digital health projects [22]. These World Health Organization guidelines may help inform IRB pathways to accelerate review of implementation science projects involving digital health and to ensure that digital health products are meeting essential standards.

In some cases, app-based interventions may be assessed as low risk. If so, such a project could fall into the expedited review category. Several studies have shown the benefit of digital health technologies in clinical outcomes of patients [22, 23]. Digital tools also enable more scalable, standardized, and low-burden delivery of implementation strategies. Using these technologies, implementation science studies can collect immense amounts of data with high temporal resolution. This capacity allows continuous monitoring of implementation outcomes and more rapid, iterative refinement of implementation strategies.

Given these complex oversight requirements, it is arguably necessary for IRBs and associated governance bodies at clinical institutions to adopt dedicated review pathways for implementation science projects that use digital health tools. Such pathways should account for valid concerns of patient privacy and data safety while also considering the benefit of digital health integration into patient care and the sustainability of such projects. Doing so would ensure patients have access to critically reviewed digital health products that may have a significant benefit to their health.

## Optimizing IRB and Regulatory Oversight to Advance Implementation Science

The following are suggestions to move the field forward.

1. Streamlined IRB approval pathway for low-risk implementation science projects. Institutions could create a distinct IRB approval pathway for low-risk implementation science projects. Many implementation science studies involve prompting clinicians to adopt or switch patients to established standards of care, or testing implementation strategies rather than novel clinical interventions, and therefore pose minimal additional risk to patients. Creating a tailored pathway for these projects would reduce unnecessary administrative burden, accelerate timelines, and better align oversight intensity with actual risk. Such a pathway could include predefined criteria for eligibility: the intervention being an evidence-based clinical practice, the only risk being to the administrative workflow, and the data being routine electronic health record or clinical performance metrics. If research satisfied these criteria, it could undergo expedited or delegated review while still maintaining ethical protections and engagement of community stakeholders to ensure appropriate oversight, trust, and buy-in.
2. Improved processes to differentiate between human subjects research and implementation science/quality improvement projects. Clearer institutional processes are needed to consistently distinguish human subjects research from quality improvement and implementation science activities, as misclassification can lead to research approval delays and in some cases overregulation. The use of a consensus checklist, recognized across organizations, could help investigators and IRB staff systematically assess which category a project may best fit under. These checklists would include items to help distinguish a low-risk implementation science project from one that should undergo more rigorous IRB oversight. These categories would be like those in institutional checklists, such as study purpose, design, risk, and endpoints [6]. Importantly, these processes should explicitly acknowledge that some projects fall into gray areas and encourage early consultation with the IRB rather than penalizing uncertainty. Effective differentiation will also require targeted training for IRB employees on the principles, designs, and goals of implementation science, which are often distinct from traditional clinical research paradigms.
3. Standardized methodology to integrate digital health into implementation science initiatives. To support the growing role of digital health in implementation science, institutions could develop standardized methodologies and coordinated review pathways that streamline evaluation across domains such as data security, project design, and vendor assessment. Rather than requiring separate reviews by multiple disconnected teams, a unified review process would improve efficiency and reduce duplication. Direct integration of similar implementation science digital projects into existing electronic health records or institutional technology platforms should be encouraged, particularly for low-risk activities like A/B testing of implementation strategies that may not require full IRB review. Additionally, developing standard forms and agreements for review that are recognized across organizations would significantly facilitate multi-center studies. These changes would enable more rapid, secure, and scalable digital implementation research while preserving data protection and regulatory compliance. The Food and Drug Administration has recently begun to implement new approval pathways for digital health tools, such as clinical decision software and consumer wearables, emphasizing the benefit of faster innovation in this space.

## Conclusions

There is a tremendous opportunity to accelerate implementation science to address the large burden of cardiometabolic disease worldwide, and innovative collaborative efforts will be needed to fulfill the potential [23]. Currently, there is no IRB approval pathway that is unique for implementation science projects. Current pathways rely on fitting implementation science projects under the binary pathway of human subjects research or quality improvement projects. By aligning oversight of IRB and regulation with low clinical risk and the pragmatic goals of implementation research, institutions can accelerate study approvals while maintaining ethical and participant protections. This creates ambiguity that delays proven clinical care from reaching patients and requires unnecessary time and capital investment. We suggest the development of standardized tools to differentiate between human subjects research, implementation science, and quality improvement projects. These tools should be used to streamline IRB approval processes of low-risk implementation science projects. Such a tool could involve a standardized checklist to aid researchers in classifying their projects as human subjects research, quality improvement, or implementation science, and enhanced guidance on the integration of digital health tools in implementation science projects. These changes have the potential to facilitate the translation of evidence-based practice to routine clinical care, improving population health and saving lives.

## Key References

- Bauer MS, Kirchner J. Implementation science: What is it and why should I care? Psychiatry Res. 2020 Jan;283:112376. doi: 10.1016/j.psychres.2019.04.025.
  - ○This review summarizes what implementation science is, its history, and its role in biomedical research.
- Powell BJ, Waltz TJ, Chinman MJ, Damschroder LJ, Smith JL, Matthieu MM, Proctor EK, Kirchner JE. A refined compilation of implementation strategies: results from the Expert Recommendations for Implementing Change (ERIC) project. Implement Sci. 2015 Feb 12;10:21. doi: 10.1186/s13012-015-0209-1.
  - ○Implementation science had been conceptualized and practiced before this study, yet was often inconsistent in terminology and study. The ERIC study sought to compile and standardize the language used in implementation science studies.
- “Quality improvement (QI) determination worksheet.” [Online]. Available: https://www.hopkinsmedicine.org/-/media/institutional-review-board/documents/QI_determination_worksheet.
  - ○Researchers often face a difficult time determining whether they should submit their implementation science project through the human subjects research IRB pipeline or quality improvement expedited pathway. This checklist published by the Johns Hopkins School of Medicine IRB attempts to aid this decision while acknowledging the ambiguity of the process.
- Fonarow GC, Albert NM, Curtis AB, Stough WG, Gheorghiade M, Heywood JT, McBride ML, Inge PJ, Mehra MR, O'Connor CM, Reynolds D, Walsh MN, Yancy CW. Improving evidence-based care for heart failure in outpatient cardiology practices: primary results of the Registry to Improve the Use of Evidence-Based Heart Failure Therapies in the Outpatient Setting (IMPROVE HF). Circulation. 2010 Aug 10;122(6):585-96. doi: 10.1161/CIRCULATIONAHA.109.934471.
  - ○The IMPROVE HF study was an early implementation science project in the cardiology space that sought to improve use of heart failure GDMT
- Rao VN, Kaltenbach LA, Granger BB, Fonarow GC, Al-Khalidi HR, Albert NM, Butler J, Allen LA, Lanfear DE, Ariely D, Miller JM, Brodsky MA, Lalonde TA, Lafferty JC, Granger CB, Hernandez AF, Devore AD. The Association of Digital Health Application Use With Heart Failure Care and Outcomes: Insights From CONNECT-HF. J Card Fail. 2022 Oct;28(10):1487-1496. doi: 10.1016/j.cardfail.2022.07.050.
  - ○This study used mobile applications to improve HFrEF care. Results showed an improvement in adherence to GDMT and clinical outcomes.

## Data Availability

No datasets were generated or analyzed for this review manuscript.

## References

[1] Khan S, Chambers D, Neta G. Revisiting time to translation: implementation of evidence-based practices (EBPs) in cancer control. Cancer Causes Control. 2021;32(3):221–30. 10.1007/s10552-020-01376-z. 33392908

[2] Subbiah V. The next generation of evidence-based medicine. Nat Med. 2023;29(1):49–58. 10.1038/s41591-022-02160-z. 36646803

[3] Machline-Carrion MJ, Soares RM, Damiani LP, Campos VB, Sampaio B, Fonseca FH, Izar MC, Amodeo C, Pontes-Neto OM, Santos JY, Gomes SPDC, Saraiva JFK, Ramacciotti E, Barros E, Silva PGM, Lopes RD, Brandão da Silva N, Guimarães HP, Piegas L, Stein AT, Berwanger O, BRIDGE Cardiovascular Prevention Investigators. Effect of a Multifaceted Quality Improvement Intervention on the Prescription of Evidence-Based Treatment in Patients at High Cardiovascular Risk in Brazil: The BRIDGE Cardiovascular Prevention Cluster Randomized Clinical Trial. JAMA Cardiol. 2019;4(5):408–17. 10.1001/jamacardio.2019.0649. 30942842 PMC6537802

[4] Bauer MS, Kirchner J. Implementation science: What is it and why should I care? Psychiatry Res. Jan. 2020;283:112376. 10.1016/j.psychres.2019.04.025. 31036287

[5] Powell BJ, Waltz TJ, Chinman MJ, Damschroder LJ, Smith JL, Matthieu MM, Proctor EK, Kirchner JE. A refined compilation of implementation strategies: results from the Expert Recommendations for Implementing Change (ERIC) project. Implement Sci. 2015;10:21. 10.1186/s13012-015-0209-1. 25889199 PMC4328074

[6] Quality improvement (QI). determination worksheet. [Online]. Available: https://www.hopkinsmedicine.org/-/media/institutional-review-board/documents/QI_determination_worksheet.

[7] Duran AT, Barbecho JM, Shaw K, Ye S, Ospina N, Simantiris S, Schwartz JE, Moise N. Implementing depression treatment for cardiac populations in rapidly changing contexts: Design of the hybrid effectiveness-implementation IHEART DEPCARE trial. Am Heart J. 2025;285:52–65. 10.1016/j.ahj.2025.02.009. 39978664 PMC12281099

[8] Definition of human subjects research | Grants & Funding, Accessed. Jul. 03, 2026. [Online]. Available: https://grants.nih.gov/policy-and-compliance/policy-topics/human-subjects/research.

[9] McLellan MC, Irshad M, Penny KC, Rufo M, Atwood S, Dacey H, Ireland CM, de Ferranti S, Saia T, Fisk AC, Saleeb SF. Enhanced Safety and Efficiency of Ambulatory Cardiology Admissions: A Quality Improvement Initiative. Pediatr Qual Saf. 2024;9(3):e726. 10.1097/pq9.0000000000000726. 38751893 PMC11093579

[10] Dickert NW, Spiegelman D, Blumenthal-Barby JS, Graham G, Joffe S, Kahn JM, Kass NE, Kim SYH, Kerlin MP, Langford AT, Lavery JV, Matlock DD, Fenton KN, Mensah GA. Ethical issues in implementation science: perspectives from a National Heart, Lung, and Blood Institute workshop. Implement Sci. 2024;19(1):77. 10.1186/s13012-024-01403-6. 39563405 PMC11577597

[11] de Waard D, Gainer R, Sim M, Cote C, Tremblay P, Bonnar P, Hirsch G. A beginner’s guide to implementation science. JTCVS Tech. 2025;32:96–101. 10.1016/j.xjtc.2025.05.005. 40814656 PMC12348288

[12] Morrato EH. Implementation Science: Ensuring the Return on Our Research Investment. Neurorehabil Neural Repair. 2018;32(9):762–4. 10.1177/1545968318794904. 30129390 PMC6404530

[13] van der Linden B, Dunham KM, Siegel J, Lazowick E, Bowdery M, Lamont T, Ford A. Health funders’ dissemination and implementation practices: results from a survey of the Ensuring Value in Research (EViR) Funders’ Forum. Implement Sci Commun. 2022;3(1):36. 10.1186/s43058-022-00273-7. 35351211 PMC8966333

[14] Fonarow GC, Albert NM, Curtis AB, Stough WG, Gheorghiade M, Heywood JT, McBride ML, Inge PJ, Mehra MR, O’Connor CM, Reynolds D, Walsh MN, Yancy CW. Improving evidence-based care for heart failure in outpatient cardiology practices: primary results of the Registry to Improve the Use of Evidence-Based Heart Failure Therapies in the Outpatient Setting (IMPROVE HF). Circulation. 2010;122(6):585–96. 10.1161/CIRCULATIONAHA.109.934471. 20660805

[15] Rao SV, O’Donoghue ML, Ruel M, Rab T, Tamis-Holland JE, Alexander JH, Baber U, Baker H, Cohen MG, Cruz-Ruiz M, Davis LL, de Lemos JA, DeWald TA, Elgendy IY, Feldman DN, Goyal A, Isiadinso I, Menon V, Morrow DA, Mukherjee D, Platz E, Promes SB, Sandner S, Sandoval Y, Schunder R, Shah B, Stopyra JP, Talbot AW, Taub PR, Williams MS, Management of Patients With Acute Coronary Syndromes. A Report of the American College of Cardiology/American Heart Association Joint Committee on Clinical Practice Guidelines. Circulation. 2025;151(13):e771–862. 10.1161/CIR.0000000000001309. 2025 ACC/AHA/ACEP/NAEMSP/SCAI Guideline for the. 40014670

[16] Bradley EH, Herrin J, Wang Y, Barton BA, Webster TR, Mattera JA, Roumanis SA, Curtis JP, Nallamothu BK, Magid DJ, McNamara RL, Parkosewich J, Loeb JM, Krumholz HM. Strategies for reducing the door-to-balloon time in acute myocardial infarction. N Engl J Med. 2006;355(22):2308–20. 10.1056/NEJMsa063117. 17101617

[17] Holtrop JS, Estabrooks PA, Gaglio B, Harden SM, Kessler RS, King DK, Kwan BM, Ory MG, Rabin BA, Shelton RC, Glasgow RE. Understanding and applying the RE-AIM framework: Clarifications and resources. J Clin Transl Sci. 2021;5(1):e126. 10.1017/cts.2021.789. 34367671 PMC8327549

[18] Mattison G, Canfell O, Forrester D, Dobbins C, Smith D, Töyräs J, Sullivan C. The Influence of Wearables on Health Care Outcomes in Chronic Disease: Systematic Review. J Med Internet Res. 2022;24(7):e36690. 10.2196/36690. 35776492 PMC9288104

[19] Longhurst CA, Singh K, Chopra A, Atreja A, Brownstein JS. A Call for Artificial Intelligence Implementation Science Centers to Evaluate Clinical Effectiveness. NEJM AI. Jul. 2024;1(8):AIp2400223. 10.1056/AIp2400223.

[20] Rao VN, Kaltenbach LA, Granger BB, Fonarow GC, Al-Khalidi HR, Albert NM, Butler J, Allen LA, Lanfear DE, Ariely D, Miller JM, Brodsky MA, Lalonde TA, Lafferty JC, Granger CB, Hernandez AF, Devore AD, Durham, North Carolina; Los Angeles, California; Cleveland, Ohio; Jackson, Misssissippi, Aurora, Colorado; Detroit, Michigan; Ewa Beach, Hawaii; Staten Island, New York. The Association of Digital Health Application Use With Heart Failure Care and Outcomes: Insights From CONNECT-HF. J Card Fail. 2022;28(10):1487–96. 10.1016/j.cardfail.2022.07.050. 35905867

[21] Office for Human Research Protections (OHRP) IRB Considerations on the use of artificial intelligence in human subjects research. Available: https://www.hhs.gov/ohrp/sachrp-committee/recommendations/irb-considerations-use-artificial-intelligence-human-subjects-research/index.html. Accessed 3 Jul 2026

[22] Marvel FA, Spaulding EM, Lee MA, Yang WE, Demo R, Ding J, Wang J, Xun H, Shah LM, Weng D, Carter J, Majmudar M, Elgin E, Sheidy J, McLin R, Flowers J, Vilarino V, Lumelsky DN, Bhardwaj V, Padula WV, Shan R, Huynh PP, Wongvibulsin S, Leung C, Allen JK, Martin SS. Digital Health Intervention in Acute Myocardial Infarction. Circ Cardiovasc Qual Outcomes. 2021;14(7):e007741. 10.1161/CIRCOUTCOMES.121.007741. 34261332 PMC8288197

[23] Jones LK, Gluckman TJ, Bhatt AS, Boer L, Bonaca MP, Campbell-Salome G, Davis E, Desai NR, George J, Head L, Marvel FA, Penn M, Peterson ED, Scirica BM, Shah NP, Wilemon K, Kalich BA, Martin SS. Accelerating evidence generation to implementation: Establishment of the Leading Awareness to Action through Implementation of Cardiometabolic Efforts (LATTICE) consortium. Am J Prev Cardiol. 2025;24:101358. 10.1016/j.ajpc.2025.101358. 41431600 PMC12718180

